# Supplementary material for: Diversity and Pathogenicity of Fusarium Species Associated with Stalk and Crown Rot in Maize in Northern Italy
Source: Plants (Basel). 2023 Nov 15;12(22):3857. doi: 10.3390/plants12223857 (PMC10674493; doi:10.3390/plants12223857)
Supplement: Supplementary file 1 [file plants-12-03857-s001.zip › plants-2674201-supplementary.pdf]

Table S1. Origin, host, culture and sequence GenBank accession number of *Fusarium* isolates used and generated in this study. Newly generated accession numbers are in bold.

Species	Complex	Collection	Host	Origin	*tef1-α*	*rpb2*	*calm*	*tub2*	Reference
F. acutatum	FFSC	CBS 401.97	*Cajanus cajan*	India	MW402124	MW402813	MW402458	MW402322	Yilmaz et al. (2021)
F. agapanthi	FFSC	CBS 100193	*Agapanthus praecox*	New Zealand	MW401959	MW402727	MW402363	MW402160	Yilmaz et al. (2021)
F. aglaonematis	FFSC	ZHKUCC 22-0079	*Aglaonema modestum*	China	ON330439	ON330445	ON330436	ON330442	Zhang et al. (2022)
F. ananatum	FFSC	CBS 118516	*Ananas comosus*	South Africa	LT996091	LT996137	MW402376	MN534089	Yilmaz et al. (2021)
F. andiyazi	FFSC	CBS 119856	Sorghum grain	Ethiopia	MN533989	MN534286	MN534174	MN534081	Yilmaz et al. (2021)
F. annulatum	FFSC	CBS 115.97	*Dianthus caryophyllus*	Italy	MW401973	MW402785	MW402373	MW402173	Yilmaz et al. (2021)
F. annulatum	FFSC	CBS 133.95	*Dianthus caryophyllus*	The Netherlands	MW402040	MW402743	MW402407	MW402239	Yilmaz et al. (2021)
F. annulatum	FFSC	CBS 135.95	*Dianthus caryophyllus*	The Netherlands	MW402043	MW402745	MW402408	MW402242	Yilmaz et al. (2021)
F. annulatum	FFSC	2.1	*Zea mays*	Italy	**OR565982**	**OR566043**	**OR566020**	**OR566004**	This study
F. annulatum	FFSC	2.2	*Zea mays*	Italy	**OR565983**	**OR566044**	**OR566021**	**OR566005**	This study
F. annulatum	FFSC	9	*Zea mays*	Italy	**OR565984**	**OR566045**	**OR566022**	**OR566006**	This study
F. annulatum	FFSC	10.1	*Zea mays*	Italy	**OR565985**	**OR566046**	**OR566023**	**OR566007**	This study
F. annulatum	FFSC	10.2	*Zea mays*	Italy	**OR565986**	**OR566047**	**OR566024**	**OR566008**	This study
F. annulatum	FFSC	55	*Zea mays*	Italy	**OR565987**	**OR566048**	**OR566025**	**OR566009**	This study
F. anthophilum	FFSC	CBS 108.92	*Hippeastrum* leaf	The Netherlands	MW401965	MW402783	MW402368	MW402166	Yilmaz et al. (2021)
F. aquaticum	FFSC	LC7502	Water	China	MW580448	MW474394	MW566275	MW533730	Wang (2022)
F. awaxy	FFSC	CBS 119831	Environmental	New Guinea	MN534056	MN534237	MN534167	MN534108	Yilmaz et al. (2021)
F. babinda	FFSC	NRRL 25539	Unknown	Unknown	KU171718	KU171698	KU171418	KU171778	O' Donnell et al. (2013)
F. bactridioides	FFSC	CBS 100057	*Cronartium conigenum* on *Pinus leiophylla*	USA	MN533993	MN534235	MN534173	MN534112	Yilmaz et al. (2021)
F. begoniae	FFSC	CBS 452.97	*Begonia elatior* hybrid	Germany	MN533994	MN534243	MN534163	MN534101	Yilmaz et al. (2021)

Species	Complex	Collection	Host	Origin	*tef1-α*	*rpb2*	*calm*	*tub2*	Reference
F. braichiariae	FFSC	CML 3163	*Brachiaria decumbens*	Brazil	MT901349	MT901315	-	MT901322	Moreira Costa et al. (2021)
F. brevicatenulatum	FFSC	CBS 404.97	*Striga asiatica*	Madagascar	MN533995	MN534295	MT010979	MN534063	Yilmaz et al. (2021)
F. bulbicola	FFSC	NRRL13618	*Nerine bowdenii* bulb	The Netherlands	KF466415	MW402767	MW402450	KF466437	Yilmaz et al. (2021)
F. caapi	FFSC	LLC3556	*Sorghum*	Ethiopia	OP486950	OP486519	OP485837	-	Lombard et al. (2022)
F. callistephi	FOSC	CBS 187.53	*Callistephus chinensis*	The Netherlands	MH484966	MH484875	MH484693	MH485057	Lombard et al. (2019)
F. carminascens	FOSC	CPC 25792	*Zea mays*	South Africa	MH485025	MH484934	MH484752	MH485116	Lombard et al. (2019)
F. casha	FFSC	PPRI20462	*Amaranthus cruentus*	South Africa	MF787262	-	-	MF787256	Vermeulen et al. (2021)
F. chinhoyiense	FFSC	NRRL 25221	*Zea mays*	Zimbabwe	MN534050	MN534262	MN534196	MN534082	Yilmaz et al. (2021)
F. chuoi	FFSC	CPC 39664	Unknown	Unknown	OK626308	OK626302	OK626304	OK626310	Yilmaz et al. (2021)
F. circinatum	FFSC	CBS 405.97	*Pinus radiata*	USA	MN533997	MN534252	MN534199	MN534097	Yilmaz et al. (2021)
F. coicis	FFSC	RBG5368	*Coix gasteenii*	Australia	KP083251	KP083274	LT996178	LT996115	Laurence et al. (2015)
F. commune	FNSC	NRRL 28387	*Dianthus caryophyllus*	The Netherlands	HM057338	JX171638	KU171420	AY329043	Han et al. (2023)
F. commune	FNSC	LC18507	*Zea mays*	China	OQ125095	OQ125101	-	-	Han et al. (2023)
F. commune	FNSC	LC18486	*Zea mays*	China	OQ125094	OQ125100	-	-	Han et al. (2023)
F. commune	FNSC	LC18652	*Zea mays*	China	OQ125093	OQ125099	-	-	Han et al. (2023)
F. commune	FNSC	LC18609	*Zea mays*	China	OQ125092	OQ125098	-	-	Han et al. (2023)
F. commune	FNSC	LC18583	*Zea mays*	China	OQ125097	OQ125103	-	-	Han et al. (2023)
F. commune	FNSC	LC18568	*Zea mays*	China	OQ125096	OQ125102	-	-	Han et al. (2023)
F. commune	FNSC	DB19LUG07	*Zea mays*	Italy	**MW419921**	**MW419923**	**OR566042**	**OR566011**	Mezzalama et al. (2021), This study
F. commune	FNSC	24	*Zea mays*	Italy	**OR565988**	**OR566049**	**OR566026**	**OR566010**	This study
F. concentricum	FFSC	CBS 450.97	*Musa sapientum*	Costa Rica	AF160282	JF741086	MW402467	MW402334	Yilmaz et al. (2021)
F. contaminatum	FOSC	CBS 111552	*Pasteurized fruit juice*	The Netherlands	MH484991	MH484900	MH484718	MH485082	Lombard et al. (2019)

Species	Complex	Collection	Host	Origin	*tef1-α*	*rpb2*	*calm*	*tub2*	Reference
F. cugenangense	FOSC	CBS 620.72	*Crocus* sp.	Germany	MH484970	MH484879	MH484697	MH485061	Lombard et al. (2019)
F. cugenangense	FOSC	CBS 130308	*Human toe nail*	New Zealand	MH485011	MH484920	MH484738	MH485102	Lombard et al. (2019)
F. cugenangense	FOSC	CBS 131393	*Vicia faba*	Australia	MH485019	MH484928	MH484746	MH485110	Lombard et al. (2019)
F. cugenangense	FOSC	CBS 130304	*Gossypium barbadense*	China	MH485012	MH484921	MH484739	MH485103	Lombard et al. (2019)
F. cugenangense	FOSC	36	*Zea mays*	Italy	**OR565989**	**OR566050**	**OR566027**	-	This study
F. curculicola	FFSC	PPRI20464	*Amaranthus cruentus*	South Africa	MF787267	MN605063	-	MF787259	Vermeulen et al. (2021)
F. curvatum	FOSC	CBS 247.61	*Matthiola incana*	Germany	MH484967	MH484876	MH484694	MH485058	Lombard et al. (2019)
F. curvatum	FOSC	CBS 238.94	*Beaucarnia* sp.	The Netherlands	MH484984	MH484893	MH484711	MH485075	Lombard et al. (2019)
F. curvatum	FOSC	CBS 141.95	*Hedera helix*	The Netherlands	MH484985	MH484894	MH484712	MH485076	Lombard et al. (2019)
F. denticulatum	FFSC	CBS 406.97	*Ipomoea batatas*	Cuba	MN533999	MN534273	MN534185	MN534067	Yilmaz et al. (2021)
F. dhileepanii	FFSC	BRIP 71717	Unknown	Unknown	OK509072	OK533536	-	-	Yilmaz et al. (2021)
F. dlaminii	FFSC	CBS 481.94	Unknown	Unknown	MN534003	MN534257	MN534151	MN534139	Yilmaz et al. (2021)
F. dlaminii	FFSC	CBS 671.94	Soil	South Africa	MN534004	MN534254	MN534152	MN534136	Yilmaz et al. (2021)
F. duoseptatum	FOSC	CBS 102026	*Musa sapientum*	Malaysia	MH484987	MH484896	MH484714	MH485078	Lombard et al. (2019)
F. echinatum	FFSC	CBS 146497	Unidentified tree	South Africa	MW834273	MW834004	MW834110	MW834301	Crous et al. (2021)
F. elaeagni	FFSC	LC13629	*Elaeagnus pungens*	China	MW580468	MW474414	MW566295	MW533750	Wang (2022)
F. elaeidis	FOSC	CBS 217.49	*Elaeis* sp.	Zaire	MH484961	MH484870	MH484688	MH485052	Lombard et al. (2019)
F. elaeidis	FOSC	CBS 255.52	*Elaeis guineensis*	Unknown	MH484965	MH484874	MH484692	MH485056	Lombard et al. (2019)
F. elaeidis	FOSC	CBS 218.49	*Elaeis sp.*	Zaire	MH484962	MH484871	MH484689	MH485053	Lombard et al. (2019)
F. fabacearum	FOSC	CPC 25801	*Zea mays*	South Africa	MH485029	MH484938	MH484756	MH485120	Lombard et al. (2019)
F. fabacearum	FOSC	CPC 25802	*Glycine max*	South Africa	MH485030	MH484939	MH484757	MH485121	Lombard et al. (2019)
F. fabacearum	FOSC	CPC 25803	*Glycine max*	South Africa	MH485031	MH484940	MH484758	MH485122	Lombard et al. (2019)

Species	Complex	Collection	Host	Origin	*tef1-α*	*rpb2*	*calm*	*tub2*	Reference
F. ficicrescens	FFSC	CBS 125177	Environmental	Iran	MN534006	MN534281	MN534176	MN534071	Yilmaz et al. (2021)
F. foetens	FOSC	CBS 120665	*Nicotiana tabacum*	Iran	MH485009	MH484918	MH484736	MH485100	Lombard et al. (2019)
F. fracticaudum	FFSC	CMW:25240	*Pinus maximinoi*	Colombia	MN534009	MN534231	MN534161	MN534103	Yilmaz et al. (2021)
F. fractiflexum	FFSC	NRRL 28852	*Cymbidium* sp.	Japan	AF160288	LT575064	AF158341	AF160315	Yilmaz et al. (2021)
F. fredkrugeri	FFSC	CBS 408.97	Soil	Maryland	MW402126	MW402814	MW402461	MW402324	Yilmaz et al. (2021)
F. fujikuroi	FFSC	CBS 186.56	Unknown	Unknown	MW402108	EF470116	MW402447	MW402306	Yilmaz et al. (2021)
F. gaditjirri	FNSC	NRRL 45417	*Hetepogon triticeus*	Australia	MN193881	MN193909	KU171424	KU171784	Sandoval-Denis et al. (2018)
F. globosum	FFSC	CBS 430.97	*Zea mays* seed	South Africa	MN534013	MN534265	MN534219	MN534125	Yilmaz et al. (2021)
F. glycines	FOSC	CBS 176.33	*Linum usitatissium*	Unknown	MH484959	MH484868	MH484686	MH485050	Lombard et al. (2019)
F. gossypinum	FOSC	CBS 116611	*Gossypium hirsutum*	Ivory Coast	MH484998	MH484907	MH484725	MH485089	Lombard et al. (2019)
F. gossypinum	FOSC	CBS 116613	*Gossypium hirsutum*	Ivory Coast	MH485000	MH484909	MH484727	MH485091	Lombard et al. (2019)
F. gossypinum	FOSC	CBS 116612	*Gossypium hirsutum*	Ivory Coast	MH484999	MH484908	MH484726	MH485090	Lombard et al. (2019)
F. guttiforme	FFSC	CBS 409.97	*Ananas comosus*	Brazil	MT010999	MT010967	MT010901	MT011048	Yilmaz et al. (2021)
F. hechiense	FFSC	LC13646	*Musa nana*	China	MW580496	MW474442	MW566323	MW533775	Wang (2022)
F. hoodiae	FOSC	CBS 132474	*Hoodia gordonii*	South Africa	MH485020	MH484929	MH484747	MH485111	Lombard et al. (2019)
F. inflexum	FOSC	NRRL 20433	*Vicia faba*	Germany	AF008479	JX171583	AF158366	–	O'Donnell et al. (2013)
F. konzum	FFSC	CBS 139382	Unknown	Unknown	MW402071	MW402804	MW402418	MW402270	Yilmaz et al. (2021)
F. lactis	FFSC	CBS 420.97	*Ficus carica*	USA	MN534015	-	MN534181	MN534078	Yilmaz et al. (2021)
F. languescens	FOSC	CBS 645.78	*Solanum lycopersicum*	Morocco	MH484971	MH484880	MH484698	MH485062	Lombard et al. (2019)
F. libertatis	FOSC	CPC 25782	*Asphalatus* sp.	South Africa	MH485023	MH484932	MH484750	MH485114	Lombard et al. (2019)
F. lumajangense	FFSC	LC13652	*Arenga caudata*	China	MW580503	MW474449	MW566330	MW533782	Wang (2022)

Species	Complex	Collection	Host	Origin	*tef1-α*	*rpb2*	*calm*	*tub2*	Reference
F. lyarnte	FNSC	NRRL 54252	*Sorghum interjectum*	Australia	MN193880	MN193908	-	-	Sandoval-Denis et al. (2018)
F. madaense	FFSC	CBS 146648	*Arachis hypogaea*	Nigeria	MW402095	MW402761	MW402436	MW402294	Yilmaz et al. (2021)
F. mangiferae	FFSC	CBS 119853	*Mangifera* sp.	South Africa	MN534016	MN534270	MN534225	MN534140	Yilmaz et al. (2021)
F. marasasianum	FFSC	CMW:25512	*Pinus tecunumanii*	Colombia	MN534018	MN534249	MN534208	MN534113	Yilmaz et al. (2021)
F. mexicanum	FFSC	NRRL 47473	*Mangifera indica*	Mexico	GU737416	LR792615	GU737389	GU737308	Yilmaz et al. (2021)
F. mirum	FFSC	LLC929	*Sorghum*	Ethiopia	OP487012	OP486581	OP485896	-	Lombard et al. (2022)
F. miscanthi	FNSC	NRRL 26231	*Miscanthus sinensis*	Japan	KU171725	KU171705	KU171425	KU171785	Han et al. (2023)
F. mundagurra	FFSC	RBG5717	Soil	Australia	KP083256	KP083276	MN534214	MN534146	Yilmaz et al. (2021)
F. napiforme	FFSC	NRRL25196	*Pennisetum typhoides*	South Africa	MN193863	MN534291	MN534192	MN534085	Laraba et al. (2020)
F. nirenbergiae	FOSC	CBS 129.24	*Secale cereale*	Unknown	MH484955	MH484864	MH484682	MH485046	Lombard et al. (2019)
F. nirenbergiae	FOSC	CBS 127.81	*Chrysantemum* sp.	USA	MH484974	MH484883	MH484701	MH485065	Lombard et al. (2019)
F. nirenbergiae	FOSC	CBS 840.88	*Dianthus caryophyllus*	The Netherlands	MH484978	MH484887	MH484705	MH485069	Lombard et al. (2019)
F. nirenbergiae	FOSC	CBS 744.79	*Passiflora edulis*	Brazil	MH484973	MH484882	MH484700	MH485064	Lombard et al. (2019)
F. nirenbergiae	FOSC	1RI (Pta 1.2)	*Zea mays*	Italy	**OR565990**	**OR566051**	**OR566028**	-	This study
F. nisikadoi	FNSC	NRRL 25179	*Phyllostachys nigra*	Japan	MN193879	MN193907	-	-	Sandoval-Denis et al. (2018)
F. nygamai	FFSC	CBS 413.97	*Oryza sativa*	Morocco	MW402127	MW402815	MW402462	MW402325	Yilmaz et al. (2021)
F. odoratissimum	FOSC	CBS 794.70	*Albizzia julibrissin*	Iran	MH484969	MH484878	MH484696	MH485060	Lombard et al. (2019)
F. ophioides	FFSC	CBS 118510	*Panicum maximum*	South Africa	MN534020	MN534301	MN534201	MN534121	Yilmaz et al. (2021)
F. oxysporum	FOSC	CBS 144134	*Solanum tuberosum*	Germany	MH485044	MH484953	MH484771	MH485135	Lombard et al. (2019)
F. oxysporum	FOSC	CBS 144135	*Solanum tuberosum*	Germany	MH485045	MH484954	MH484772	MH485136	Lombard et al. (2019)
F. oxysporum	FOSC	CBS 221.49	*Camellia sinensis*	South East Asia	MH484963	MH484872	MH484690	MH485054	Lombard et al. (2019)

Species	Complex	Collection	Host	Origin	*tef1-α*	*rpb2*	*calm*	*tub2*	Reference
F. oxysporum	FOSC	CPC 25822	*Protea* sp.	South Africa	MH485034	MH484943	MH484761	MH485125	Lombard et al. (2019)
F. oxysporum sensu lato	FOSC	11	*Zea mays*	Italy	**OR565991**	**OR566052**	**OR566029**	-	This study
F. oxysporum sensu lato	FOSC	12	*Zea mays*	Italy	**OR565992**	**OR566053**	**OR566030**	-	This study
F. oxysporum sensu lato	FOSC	18	*Zea mays*	Italy	**OR565993**	**OR566054**	**OR566031**	-	This study
F. oxysporum sensu lato	FOSC	26	*Zea mays*	Italy	**OR565994**	**OR566055**	**OR566032**	-	This study
F. oxysporum sensu lato	FOSC	51	*Zea mays*	Italy	**OR565995**	**OR566056**	**OR566033**	-	This study
F. panlongense	FFSC	LC13656	*Musa nana*	China	MW580510	MW474456	MW566337	MW533789	Wang (2022)
F. paranisikadoi	FNSC	LC2824	*Zea mays*	China	MW594317	MW474550	-	MW533921	Han et al. (2023)
F. parvisorum	FFSC	CMW:25267	*Pinus patula*	Colombia	KJ541060	-	-	KJ541055	Yilmaz et al. (2021)
F. pharetrum	FOSC	CPC 30822	*Aliodendron dichotomum*	South Africa	MH485042	MH484951	MH484769	MH485133	Lombard et al. (2019)
F. phyllophilum	FFSC	NRRL13617	*Dracaena deremensis*	Italy	MN193864	KF466410	KF466333	KF466443	Laraba et al. (2020)
F. pilosicola	FFSC	NRRL 29123	*Bidens pilosa*	USA	MN534054	MN534247	MN534165	MN534098	Yilmaz et al. (2021)
F. pininemorale	FFSC	CMW:25243	*Pinus tecunumanii*	Colombia	MN534026	MN534250	MN534211	MN534115	Yilmaz et al. (2021)
F. proliferatum	FFSC	CBS 480.96	Tropical rain forest soil	Papua New Guinea	MN534059	MN534272	MN534217	MN534129	Yilmaz et al. (2021)
F. pseudoanthophilum	FFSC	CBS 745.97	*Zea mays*	Zimbabwe	MW402148	MW402820	MW402476	MW402349	Yilmaz et al. (2021)
F. pseudocircinatum	FFSC	NRRL22946	*Solanum* sp.	Ghana	AF160271	MN534277	MN534190	MN534069	O' Donnell et al. (2000)
F. pseudonygamai	FFSC	CBS 416.97	*Pennisetum typhoides*	Nigeria	MN534030	MN534283	MN534194	MN534064	Yilmaz et al. (2021)
F. ramigenum	FFSC	NRRL25208	*Ficus carica*	USA	KF466423	KF466412	MN534187	MN534145	Proctor et al. (2013)

Species	Complex	Collection	Host	Origin	*tef1-α*	*rpb2*	*calm*	*tub2*	Reference
F. sacchari	FFSC	CBS 131370	*Oryzae australiensis*	Australia	MW402031	MW402793	MW402404	MW402230	Yilmaz et al. (2021)
F. secorum	FFSC	NRRL 62593	*Beta vulgaris*	USA	KJ189225	–	KJ189235	–	Yilmaz et al. (2021)
F. siculi	FFSC	CPC 27188	*Citrus sinensis*	Italy	LT746214	LT746327	LT746189	LT746346	Sandoval-Denis et al. (2018)
F. sororula	FFSC	CMW:25513	*Pinus tecunumanii*	Colombia	MN534035	MN534246	MN534210	MN534114	Yilmaz et al. (2021)
F. sterilihyposum	FFSC	NRRL 25623	*Mangifera sp.*	South Africa	MN193869	MN193897	AF158353	AF160316	Yilmaz et al. (2021)
F. subglutinans	FFSC	CBS 215.76	*Zea mays*	Germany	MN534061	MN534241	MN534171	MN534109	Yilmaz et al. (2021)
F. succisae	FFSC	CBS 187.34	*Zostera marina*	UK	MW402109	MW402810	MW402448	MW402307	Yilmaz et al. (2021)
F. sudanense	FFSC	CBS 454.97	*Striga hermonthica*	Sudan	MN534037	MN534278	MN534179	MN534073	Yilmaz et al. (2021)
F. tardichlamydosporum	FOSC	CBS 102028	*Musa sapientum*	Malaysia	MH484988	MH484897	MH484715	MH485079	Lombard et al. (2019)
F. temperatum	FFSC	CBS 135538	Pulmonary infection (human)	Mexico	MN534039	MN534239	MN534168	MN534111	Yilmaz et al. (2021)
F. terricola	FFSC	CBS 483.94	Soil	Australia	MN534042	LT996156	MN534189	MN534076	Yilmaz et al. (2021)
F. thapsinum	FFSC	CBS 539.79	Man, white grained mycetoma	Italy	MW402140	MW402818	MW402472	MW402340	Yilmaz et al. (2021)
F. tjaetaba	FFSC	RBG5361	*Sorghum interjectum*	Australia	KP083263	KP083275	LT996187	GU737296	Laurence et al. (2015)
F. triseptatum	FOSC	CBS 258.50	*Ipomoea batatas*	USA	MH484964	MH484873	MH484691	MH485055	Lombard et al. (2019)
F. tupiense	FFSC	NRRL 53984	*Mangifera indica*	Brazil	GU737404	LR792619	GU737377	GU737350	Yilmaz et al. (2021)
F. udum	FFSC	NRRL22949	Unknown	Unknown	AF160275	LT996172	MW402442	U34433	O' Donnell et al. (2000)
F. verticillioides	FFSC	CBS 116665	*Solanum lycopersicum*	Unknown	MW401976	MW402825	MW402375	MW402176	Yilmaz et al. (2021)
F. verticillioides	FFSC	CBS 125.73	*Trichosanthes dioica*	India	MW402012	MW402791	MW402392	MW402212	Yilmaz et al. (2021)
F. verticillioides	FFSC	CBS 167.87	*Pinus* seed	USA	MW402101	-	MW402441	MW402300	Yilmaz et al. (2021)
F. verticillioides	FFSC	CBS 447.95	*Asparagus*	Unknown	MW402133	MW402770	MW402466	MW402332	Yilmaz et al. (2021)

Species	Complex	Collection	Host	Origin	*tef1-α*	*rpb2*	*calm*	*tub2*	Reference
F. verticillioides	FFSC	CBS 531.95	*Zea mays*	Unknown	MW402136	MW402771	MW402468	MW402336	Yilmaz et al. (2021)
F. verticillioides	FFSC	CBS 131389	Environmental	Australia	KU711695	KU604226	MN534193	KU603857	Yilmaz et al. (2021)
F. verticillioides	FFSC	CBS 734.97	*Zea mays*	Germany	MW402146	EF470122	AF158315	MW402346	Yilmaz et al. (2021)
F. verticillioides	FFSC	8.2	*Zea mays*	Italy	**OR565996**	**OR566057**	**OR566034**	**OR566012**	This study
F. verticillioides	FFSC	35.1.4	*Zea mays*	Italy	**OR565997**	**OR566058**	**OR566035**	**OR566013**	This study
F. verticillioides	FFSC	56.1.2	*Zea mays*	Italy	**OR565998**	**OR566059**	**OR566036**	**OR566014**	This study
F. verticillioides	FFSC	56.2.2	*Zea mays*	Italy	**OR565999**	**OR566060**	**OR566037**	**OR566015**	This study
F. verticillioides	FFSC	56.2.3	*Zea mays*	Italy	**OR566000**	**OR566061**	**OR566038**	**OR566016**	This study
F. verticillioides	FFSC	56.2.4	*Zea mays*	Italy	**OR566001**	**OR566062**	**OR566039**	**OR566017**	This study
F. verticillioides	FFSC	56.2.5	*Zea mays*	Italy	**OR566002**	**OR566063**	**OR566040**	**OR566018**	This study
F. verticillioides	FFSC	57.2.1	*Zea mays*	Italy	**OR566003**	**OR566064**	**OR566041**	**OR566019**	This study
F. veterinarium	FOSC	CBS 109898	*Shark peritoneum*	The Netherlands	MH484990	MH484899	MH484717	MH485081	Lombard et al. (2019)
F. volatile	FFSC	CBS 143874	Human bronchoalveolar lavage fluid	French Guiana	LR596007	LR596006	MK984595	LR596008	Yilmaz et al. (2021)
F. werrikimbe	FFSC	CBS 125535	*Sorghum leiocladum*	Australia	MW928846	MN534304	MN534203	MN534104	Yilmaz et al. (2021)
F. xylarioides	FFSC	NRRL25486	*Coffea* trunk	Ivory Coast	MN193874	HM068355	MW402455	AY707118	Laraba et al. (2020)
F. xyrophilum	FFSC	NRRL 62710	*Xyris* spp.	Guyana	MN193875	MN193903	–	–	Yilmaz et al. (2021)
